# Blood flow restriction training attenuates changes in local muscle endurance: At odds with previous work?

**DOI:** 10.1113/EP091924

**Published:** 2024-07-19

**Authors:** William B. Hammert, Ryo Kataoka, Yujiro Yamada, Jun Seob Song, Jeremy P. Loenneke

**Affiliations:** ^1^ Kevser Ermin Applied Physiology Laboratory Department of Health, Exercise Science, and Recreation Management University of Mississippi University Mississippi USA

**Keywords:** blood flow restriction, endurance, exercise

Ida and Sasaki (2024) reported that exercising to failure without blood flow restriction resulted in greater increases in local muscle endurance than exercising to failure with blood flow restriction. The authors attributed their results (of their second experiment) to the differences in total exercise volume (i.e., load multiplied by the total number of repetitions over the training period) between conditions, and provided a correlation for support (Ida & Sasaki, 2024). Their correlation, however, appears to count each individual twice (i.e., *n *= 8 with 16 dots), which would violate the assumption of independence. Dr. Wernbom (2024) recently provided a viewpoint on Ida and Sasaki's study. We agree that more research is needed to better understand how low load resistance exercise with blood flow restriction impacts the mechanisms underlying improvements in muscle endurance. However, we also believe it is important to discuss Ida and Sasaki's findings in the context of the existing literature. More specifically, we believe their observation that blood flow restriction attenuated changes in local muscle endurance, and their explanation for such, should be acknowledged as being at odds with published studies.

Table 1 summarizes the characteristics and results of 10 blood flow restriction training studies that examined changes in local muscle endurance using pre‐ to post‐intervention changes in the number of repetitions to failure (i.e., in a manner similar to that of Ida and Sasaki (2024)). In their introduction, Ida and Sasaki (2024) pointed out that, to their knowledge, the impact of blood flow restriction on exercise volume, muscle endurance adaptations, and their association has not been studied systematically. Over 10 years ago, however, Kacin and Strazar (2011) reported that exercising to failure with blood flow restriction improved local muscle endurance to a greater extent than a repetition‐matched, low load training protocol performed without blood flow restriction (Table 1). In their discussion, Ida and Sasaki (2024) acknowledged the work of Kacin and Strazar (2011), and suggested that the discrepancies between studies highlight the importance of exercise volume for improving muscle endurance capacity. A few years after the Kacin and Strazar study was published, Fahs et al. (2015) compared muscular adaptations between low load resistance training with and without blood flow restriction in middle‐aged men and women (i.e., 42–62 years). In comparison to Kacin and Strazar's study, Fahs et al.’s study was unique in that both training protocols exercised to failure. Fahs et al. (2015) observed similar increases in local muscle endurance, despite the fact that blood flow restriction significantly reduced the total exercise training volume. Two other studies (Buckner et al., 2020; Jessee et al., 2018) have demonstrated that performing very low load training (to or near failure) with and without lower blood flow restriction pressures led to similar increases in local muscle endurance. Importantly, the application of blood flow restriction resulted in fewer repetitions (and thus, total training volume) being performed across an 8‐week training period (Buckner et al., 2020; Jessee et al., 2018). Indeed, in the study of Jessee et al. (2018), the application of a higher blood flow restriction pressure resulted in fewer repetitions (i.e., lower total training volume) being performed, yet actually enhanced local muscle endurance adaptations over and above very low load training (to or near failure) without blood flow restriction. Additional data have shown that higher applied pressures result in lower total training volumes, but similar increases in local muscular endurance compared to lower applied pressure (Buckner et al., 2020; Counts et al., 2016; Morley et al., 2021).

**TABLE 1:** The characteristics and results of 10 blood flow restriction training studies that examined changes in local muscle endurance using pre‐ to post‐intervention changes in the number of repetitions to failure.

Reference	Study Design	Exercise Training protocols	Muscle Endurance Test	Results
Kacin & Strazar (2011)	4 wks; 4×/wk: Within‐subject Unilateral KE 1 s Con, 1 s Ecc	A: 4 × F, 15% MVC + I‐BFR (230 mmHg); 2 min B: 4 × rep‐matched to ‘A’, 15% MVC; 2 min	Unilateral KE, 15% Pre‐MVC + BFR (230 mmHg) Unilateral KE, 15% Pre‐MVC	↑ A, B ***A > B*** ↑ A, B ***A > B***
Fahs et al. (2015)	6 wks; 3×/wk: Within‐subject Unilateral KE 1.5 s Con, 1.5 s Ecc	A: 2–4 × F, 30% 1RM + BFR (goal of 50%–80% AOP, progressed across weeks); 1 min B: 2–4 × F, 30% 1RM; 1 min	Unilateral KE, 30% Post‐1RM 1.5 s Con, 1.5 s Ecc	↑ A, B ***A ↔ B***
Counts et al. (2016)	8 wks; 2–3×/wk: Within‐subject Unilateral EF 1 s Con, 1 s Ecc	A: 1 × 30, 3 × 15, 30% 1RM + BFR (40% AOP); 30 s B: 1 × 30, 3 × 15, 30% 1RM + BFR (90% AOP); 30 s	Unilateral EF, 30% Post‐1RM 1 s Con, 1 s Ecc	↑ A, B ***A ↔ B***
Jessee et al. (2018)	8 wks; 2×/wk: Within‐between subject Unilateral KE 1 s Con, 1 s Ecc	A: 4 × 90 or F, 15% 1RM; 30 s B: 4 × 90 or F, 15% 1RM + BFR (40% AOP); 30 s C: 4 × 90 or F, 15% 1RM + BFR (80% AOP); 30 s D: 4 × F, 70% 1RM; 90 s	Unilateral KE, 42.5% Pre‐1RM 1 s Con, 1 s Ecc	↑ A, B, C, D ***C > A, D; B ↔ C***
Groennebaek et al. (2018)	6 wks; 3×/wk: Between‐subject Bilateral KE 1.5 s Con, 1.5 s Ecc	A: 4 × F, ∼30% 1RM + BFR (50% AOP); 30 s B: 4 × 12, ∼70% 1RM; 3 min C: Non‐exercise control	Bilateral KE, ∼30% Post‐1RM 1.5 s Con, 1.5 s Ecc	↑ A; ↔ B, C ***A > C; A ↔ B***
Sieljacks et al. (2019)	8 wks; 2–3×/wk: Within‐subject Unilateral KE 1 s Con, 1 s Ecc	A: 4 × F, ∼25% 1RM + BFR (40% AOP); 45 s B: 4 × 75% reps of ‘A’, ∼25% 1RM + BFR (40% AOP); 45 s	Unilateral KE, ∼25% Post‐1RM 1 s Con, 1 s Ecc	A ↔ B ***A ↔ B***
de Lemos Muller et al. (2019)	6 wks; 3×/wk: Between‐subject Bilateral EF, KE Con, NR; Ecc, NR	A: 4 × 8, 80% 1RM; 2 min B: 4 × 22, 30% 1RM + BFR (20 mmHg ± SBP); 2 min	Bilateral EF, 60% Post‐1RM 2 s Con, 2 s Ecc Bilateral KE, 60% Post‐1RM 2 s Con, 2 s Ecc	↑ A, B ***A ↔ B*** ↑ A, B ***A ↔ B***
Rodrigues Neto et al. (2019)	6 wks; 2×/wk: Between‐subject BP, FP, EE, EF 1.5 s Con, 1.5 s Ecc	A: 4 × 15, 20% 1RM + BFR (80% AOP); 30 s B: 4 × 15, 20% 1RM + I‐BFR (80% AOP); 30 s C: 4 × 15, 20% 1RM; 30 s	BP, 40%; 1RM, (% pre or post NR) 1.5 s Con, 1.5 s Ecc FP, 40%; 1RM, (% pre or post NR) 1.5 s Con, 1.5 s Ecc EE, 40%; 1RM, (% pre or post NR) 1.5 s Con, 1.5 s Ecc EF, 40%; 1RM, (% pre or post NR) 1.5 s Con, 1.5 s Ecc	↔ A, B, C ***A ↔ B ↔ C*** ↔ A, B, C ***A ↔ B ↔ C*** ↑ A; ↔ B, C ***A ↔ B ↔ C*** ↑ A, B, C ***A ↔ B ↔ C***
Buckner et al. (2020)	8 wks; 2×/wk: Within‐between subject Unilateral EF 1 s Con, 1 s Ecc	A: 4 × 90 or F, 15% 1RM; 30 s B: 4 × 90 or F, 15% 1RM + BFR (40% AOP); 30 s C: 4 × 90 or F, 15% 1RM + BFR (80% AOP); 30 s D: 4 × F, 70% 1RM; 90 s	Unilateral EF, 42.5% Pre‐1RM 1 s Con, 1 s Ecc	↑ A, B, C, D ***A ↔ B ↔ C ↔ D***
Morley et al. (2021)	8 wks, 3 ×/wk: Between‐subject Unilateral KE Con, NR; Ecc, NR	A: 3 × 10–12 + 1 × F, 20% 1RM + BFR (100% AOP); 1 min B: 3 × 10–12 + 1 × F, 20% 1RM + BFR (50% AOP); 1 min C: 3 × 10–12 + 1 × F, 70% 1RM; 1 min	Unilateral KE, 20% 1RM, (% pre or post NR) 1.5 s Con, 1.5 s Ecc	↑ A, B, C ***A ↔ B ↔ C***

*Note*: NR: information not reported. Study Design: study duration, sessions/week, within‐subject design (if applicable), exercise selection, repetition cadence (if reported). Exercise training protocols: sets × repetitions, load (% maximal strength); inter‐set rest interval. Muscle Endurance Test: exercise, load (% Pre‐ or Post‐1RM on the post‐intervention endurance test, if reported) and repetition cadence (if reported). Results: ↑ statistically significant increase from pre‐ to post‐intervention; ↓ statistically significant decrease from pre‐ to post‐intervention; ↔ no statistically significant change from pre‐ to post‐intervention. Between protocol comparisons are italicized and shown in bold: **>** statistically significant differences between exercise training protocols (e.g., **A > B** means that training protocol A demonstrated statistically significantly greater changes from pre‐ to post‐intervention compared to training protocol B); **↔** no statistically significant difference between exercise training protocols (e.g., **A ↔ B** means that training protocol A and B did not statistically change differently from pre‐ to post‐intervention). Abbreviations: 1RM, one ‐repetition maximum; AOP, arterial occlusion pressure; BP, bench press; BFR, blood flow restriction applied continuously; Con, concentric muscle action; Ecc, eccentric muscle action; EE, elbow extension; EF, elbow flexion; F, repetitions to failure; FP, front pulldown; I‐BFR, intermittent blood flow restriction; KE, knee extension; MVC, maximal voluntary contraction; reps, repetitions; RM, repetition maximum; s, seconds; wk(s), week(s).

Ida and Sasaki (2024) concluded that blood flow restriction may impair changes in local muscle endurance compared to free flow exercise (i.e., exercising without blood flow restriction), which is at odds with previous research (Table 1). They (Ida & Sasaki, 2024) attributed their findings to the differences in total training volume between conditions, which is also at odds with previous research (Buckner et al., 2020; Fahs et al., 2015; Jessee et al., 2018). We are not suggesting that Ida and Sasaki (2024) should have acknowledged and discussed all 10 studies that are presented in Table 1, as some appear to be less relevant to their current design. However, in our opinion, it would have been helpful to at least discuss the Fahs et al. (2015) study, as well as those which prescribed lower load resistance training to or near failure with and without blood flow restriction (Buckner et al., 2020; Jessee et al., 2018). The literature suggests that performing low load resistance training to (or near) failure with blood flow restriction will likely reduce total training volume compared to low load training without blood flow restriction (Buckner et al., 2020; Fahs et al., 2015; Jessee et al., 2018). Yet, apart from the study of Ida and Sasaki (2024), it does not seem as though such reductions in total training volume will attenuate changes in local muscle endurance compared to performing low load resistance training without blood flow restriction (Table 1). We acknowledge that variation exists in the training protocols used across the highlighted studies, as well as how changes in local muscle endurance have been measured (Table 1), and that each study is not without its own set of potential limitations (e.g., within‐subject unilateral training models). The discrepancies between Ida and Sasaki (2024)’s findings and the rest of the literature remain speculative, but may stem from Ida and Sasaki (2024)’s use of a one‐set exercise training protocol. Perhaps multiple sets of exercise, as used in previous studies (Table 1), provides a stimulus that is capable of eliminating the attenuation observed with single‐set blood flow restricted exercise. More research is needed to corroborate Ida and Sasaki's (2024) data and provide additional insight into how applying blood flow restriction during a single‐set exercise training protocol impacts changes in local muscle endurance, perhaps compared to single‐ and multiple‐set exercise protocols without blood flow restriction.

## AUTHOR CONTRIBUTIONS

All authors approved the final version of the manuscript and agree to be accountable for all aspects of the work in ensuring that questions related to the accuracy or integrity of any part of the work are appropriately investigated and resolved. All persons designated as authors qualify for authorship, and all those who qualify for authorship are listed.

## CONFLICT OF INTEREST

None declared.

## FUNDING INFORMATION

None.

## References

[eph13554-bib-0001] Buckner, S. L. , Jessee, M. B. , Dankel, S. J. , Mattocks, K. T. , Mouser, J. G. , Bell, Z. W. , Abe, T. , Bentley, J. P. , & Loenneke, J. P. (2020). Blood flow restriction does not augment low force contractions taken to or near task failure. European Journal of Sport Science, 20(5), 650–659. 31486720 10.1080/17461391.2019.1664640

[eph13554-bib-0002] Counts, B. R. , Dankel, S. J. , Barnett, B. E. , Kim, D. , Mouser, J. G. , Allen, K. M. , Thiebaud, R. S. , Abe, T. , Bemben, M. G. , & Loenneke, J. P. (2016). Influence of relative blood flow restriction pressure on muscle activation and muscle adaptation: Relative BFR Pressure. Muscle & Nerve, 53(3), 438–445. 26137897 10.1002/mus.24756

[eph13554-bib-0003] de Lemos Muller, C. H. , Ramis, T. R. , & Ribeiro, J. L. (2019). Effects of low‐load resistance training with blood flow restriction on the perceived exertion, muscular resistance and endurance in healthy young adults.Sport Sciences for Health, 15, 503–510.

[eph13554-bib-0004] Fahs, C. A. , Loenneke, J. P. , Thiebaud, R. S. , Rossow, L. M. , Kim, D. , Abe, T. , Beck, T. W. , Feeback, D. L. , Bemben, D. A. , & Bemben, M. G. (2015). Muscular adaptations to fatiguing exercise with and without blood flow restriction. Clinical Physiology and Functional Imaging, 35(3), 167–176. 24612120 10.1111/cpf.12141

[eph13554-bib-0005] Groennebaek, T. , Jespersen, N. R. , Jakobsgaard, J. E. , Sieljacks, P. , Wang, J. , Rindom, E. , Musci, R. V. , Bøtker, H. E. , Hamilton, K. L. , Miller, B. F. , De Paoli, F. V. & , & Vissing, K. (2018). Skeletal muscle mitochondrial protein synthesis and respiration increase with low‐load blood flow restricted as well as high‐load resistance training. Frontiers in Physiology, 9, 1796. 30618808 10.3389/fphys.2018.01796 PMC6304675

[eph13554-bib-0006] Ida, A. , & Sasaki, K. (2024). Distinct adaptations of muscle endurance but not strength or hypertrophy to low‐load resistance training with and without blood flow restriction. Experimental Physiology, 109(6), 926–938. 38502540 10.1113/EP091310 PMC11140179

[eph13554-bib-0007] Jessee, M. B. , Buckner, S. L. , Mouser, J. G. , Mattocks, K. T. , Dankel, S. J. , Abe, T. , Bell, Z. W. , Bentley, J. P. , & Loenneke, J. P. (2018). Muscle adaptations to high‐load training and very low‐load training with and without blood flow restriction. Frontiers in Physiology, 9, 1448. 30386254 10.3389/fphys.2018.01448 PMC6198179

[eph13554-bib-0008] Kacin, A. , & Strazar, K. (2011). Frequent low‐load ischemic resistance exercise to failure enhances muscle oxygen delivery and endurance capacity: Ischemic training and muscle endurance. Scandinavian Journal of Medicine & Science in Sports, 21(6), e231–e241. 21385216 10.1111/j.1600-0838.2010.01260.x

[eph13554-bib-0009] Morley, W. N. , Ferth, S. , Debenham, M. I. B. , Boston, M. , Power, G. A. , & Burr, J. F. (2021). Training response to 8 weeks of blood flow restricted training is not improved by preferentially altering tissue hypoxia or lactate accumulation when training to repetition failure. Applied Physiology, Nutrition and Metabolism, 46(10), 1257–1264. 10.1139/apnm-2020-1056 33930277

[eph13554-bib-0010] Rodrigues Neto, G. , Silva, J. , Freitas, L. , Silva, H. G. D. , Caldas, D. , Novaes, J. D. S. , & Cirilo‐Sousa, M. S. (2019). Effects of strength training with continuous or intermittent blood flow restriction on the hypertrophy, muscular strength and endurance of men. Acta Scientiarum Health Sciences, 41(1), 42273.

[eph13554-bib-0011] Sieljacks, P. , Degn, R. , Hollaender, K. , Wernbom, M. , & Vissing, K. (2019). Non‐failure blood flow restricted exercise induces similar muscle adaptations and less discomfort than failure protocols. Scandinavian Journal of Medicine & Science in Sports, 29(3), 336–347. 30475424 10.1111/sms.13346

[eph13554-bib-0012] Wernbom, M. (2024). Low‐load resistance exercise with and without blood flow restriction: Which is more effective for increasing local muscle endurance and why? Experimental Physiology, 109(6), 839–840. 38520700 10.1113/EP091872 PMC11140171

